# Collaborative and scalable training model for rural comunities of La Sierra Madre in Chiapas, México: An opportunity for global mental health in low-middel income areas

**DOI:** 10.1192/j.eurpsy.2021.1937

**Published:** 2021-08-13

**Authors:** M. Oscoz Irurozqui, F. Rodríguez-Cuevas, A. López-Salinas

**Affiliations:** 1 Psychiatry, Centro Salud Mental Milagrosa y II Ensanche, Pamplona, Spain; 2 Psychiatry, Compañeros en Salud, Jaltenango de la Paz, Mexico; 3 Psychiatry, Tecnológico de Monterrey, Monterrey, Mexico

**Keywords:** health worker, task-sharing, cost-effectiveness., global mental health

## Abstract

**Introduction:**

In Chiapas, Mexico, it is estimated that 1,356 million people suffer from depression; there are about 210 psychologists (1/24,847 people) and 4 psychiatrists (1/1,304 people). Collaborative task sharing, which engages nonspecialists in mental health care delivery, is essential to address the large global burden of mental illness. The collaborative care model (CoCM), a specific type of task-sharing strategy, incorporates a team-based approach with: a primary care provider (PCP); a behavioral health professional, who is the care manager (CM); and a consulting psychiatrist. CoCM has shown improved outcomes for both mental and general health, expanded access to care, and cost-effectiveness.

**Objectives:**

Our objective is to implement one of the arms of the phased model and CoCM, through supervision and training of health professionals not specialists in Mental Health, in different clinical spaces of community primary care, by specialists.

**Methods:**

We implemented a training program taught by psychiatrists and psychologists for health workers in communities of La Sierra Madre in Chiapas, which includes: training of intern nurses, training and supervision of intern doctors and on-site supervision and training of community mental health workers (CMHW); all undergoing a process of monitoring, evaluation and quality.

**Results:**

Of the patients that were treated (202; 89% women), more than 80% had a diagnosis of anxiety and depression. The most notable clinical improvement (measured with the PHQ-9 depression scale) occurred in the intervention group of CMHW + interns (reduction PHQ-9 58%).

**Conclusions:**

This strategy seems acceptable to address the large gaps in the availability of mental health providers in low-income countries.

**Disclosure:**

No significant relationships.

